# Dense granule proteins: key mediators of *Toxoplasma gondii* pathogenesis and therapeutic targets

**DOI:** 10.3389/fmed.2026.1770993

**Published:** 2026-05-04

**Authors:** Ruiming Zeng, Hammad Bacha, Shams Uz Zaman, Mohammed Abohashrh, Rasha Alonaizan, Khalid J. Alzahrani, Khalaf F. Alsharif, Fuad M. Alzahrani, Abdul Qadeer, Qingming Fu

**Affiliations:** 1 Hunan Provincial Key Laboratory of the Research and Development of Novel Pharmaceutical Preparation, Changsha Medical University, Changsha, China; 2 Department of Burn and Plastic Surgery, The Third Xiangya Hospital of Central South University, Changsha, Hunan, China; 3 Changsha Medical University, Changsha, China; 4 Department of Basic Medical Sciences, College of Applied Medical Sciences, King Khalid University, Abha, Saudi Arabia; 5 Department of Zoology, College of Science, King Saud University, Riyadh, Saudi Arabia; 6 Department of Clinical Laboratories Sciences, College of Applied Medical Sciences, Taif University, Taif, Saudi Arabia; 7 School of Medical Sciences, Shandong Xiehe University, Jinan, Shandong, China; 8 Laboratory of Pathogenic Biology and Immunology, Changsha Medical University, Changsha, China

**Keywords:** dense granule proteins, immune modulation, parasitophorous vacuole, pathogenesis, *Toxoplasma gondii*

## Abstract

*Toxoplasma gondii* (*T. gondii*) is an apicomplexan parasite that infects most warm-blooded animals, including approximately one-third of the human population worldwide. Dense granule proteins (GRAs), secreted from specialized organelles and trafficked to multiple compartments, including the parasitophorous vacuole (PV), parasitophorous vacuole membrane (PVM), host cytosol, and host cell nucleus following host cell invasion, have emerged as critical mediators of host–parasite interactions. This review provides a comprehensive analysis of our current knowledge of GRA proteins in toxoplasmosis pathogenesis and establishes a framework for developing novel interventions against this globally significant parasitic disease. Key GRA proteins form pore complexes that regulate PVM permeability, while GRA effectors manipulate host immunity to evade invasion. GRA proteins are essential for tissue cyst formation and bradyzoite differentiation, enabling lifelong chronic infection. The exceptional immunogenicity of GRA antigens has positioned them as promising candidates for diagnostic applications and vaccine development. Additionally, GRA proteins are attractive therapeutic targets through either the direct inhibition of effector-host protein interactions or by the disruption of effector export.

## 1 Introduction

**Figure 1:**
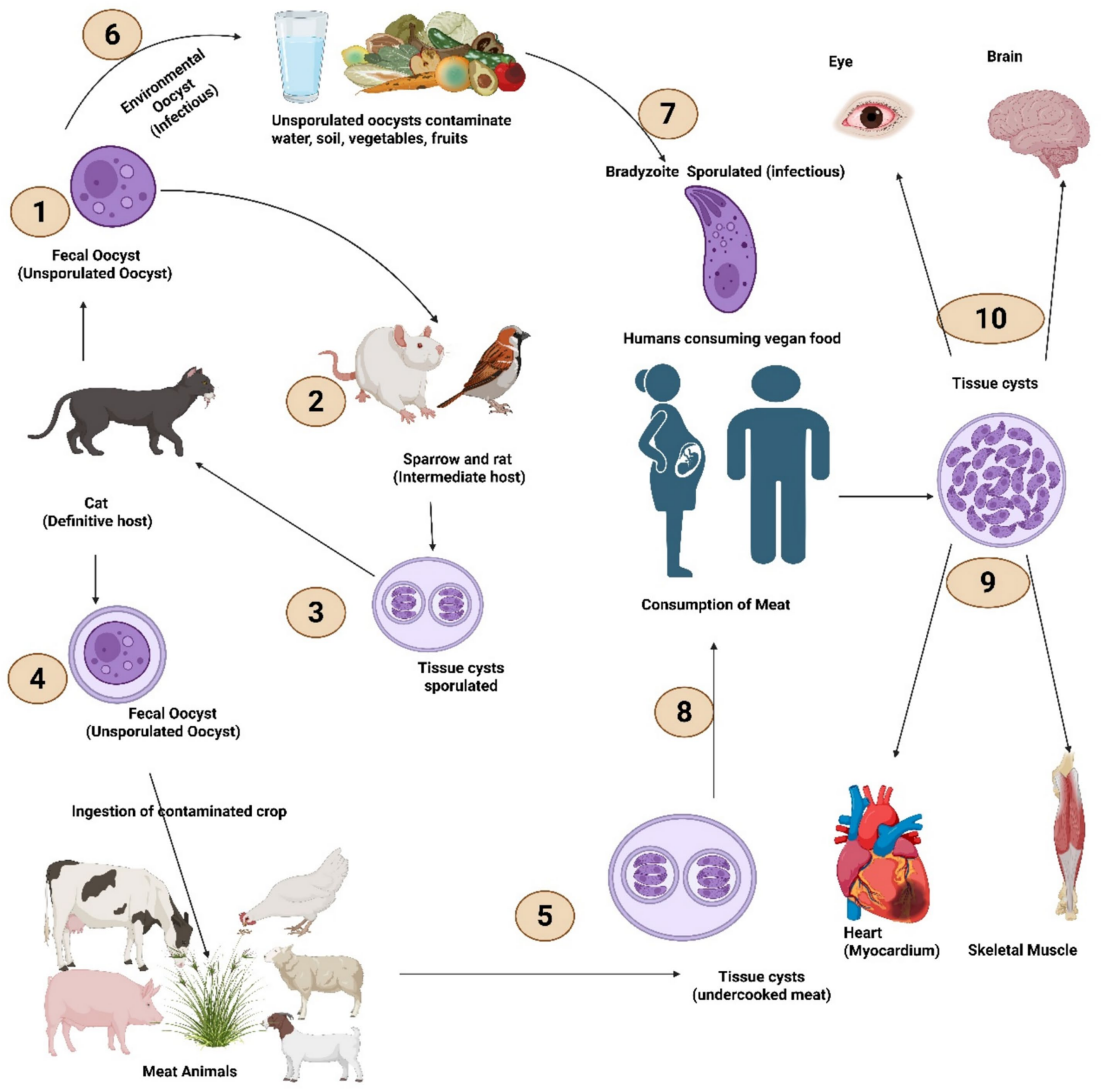
Life cycle of *T. gondii* showing transmission pathways between definitive and intermediate hosts. The life cycle of *T. gondii* involves both sexual reproduction in the definitive host and asexual proliferation in intermediate hosts. (1) Cats, the definitive host, shed unsporulated fecal oocysts in the environment. (2) Intermediate hosts, including sparrows and rodents, become infected through ingestion of these oocysts from contaminated environments. (3) Within intermediate hosts, parasites differentiate into sporulated tissue cysts containing bradyzoites, which persist in host tissues. (4) Cats become reinfected by preying on infected intermediate hosts harboring tissue cysts, subsequently shedding new unsporulated oocysts in feces. (5) Meat animals (cattle, poultry, pigs, sheep, and goats) acquire infection through ingestion of oocysts from contaminated crops and environments, developing tissue cysts in their tissues. (6) Sporulated oocysts persist in the environment as infectious forms, contaminating water, soil, vegetables, and fruits. (7) Humans consuming contaminated vegan food ingest sporulated oocysts or bradyzoites, leading to infection. (8) Humans also acquire infection through consumption of undercooked meat containing tissue cysts. (9) In infected humans, tissue cysts localize to the heart (myocardium) and skeletal muscle. (10) Tissue cysts additionally establish in the eye and brain, where they may cause ocular and neurological manifestations. Created with BioRender.com.

*Toxoplasma gondii* (*T. gondii*) causes toxoplasmosis, a disease of significant public health and veterinary importance due to its capacity to infect diverse host species and establish lifelong persistent infections (1, 2). The clinical manifestations of toxoplasmosis vary among hosts, influenced by host immune-related genes and parasite virulence determinants, with particular attention to *Toxoplasma*-associated ileitis and encephalitis (3). The parasite exhibits a complex life cycle requiring both definitive and intermediate hosts to complete its sexual and asexual reproduction cycles (Figure 1) (4, 5).

Members of the family Felidae, particularly domestic cats, are the only known definitive hosts in which *T. gondii* undergoes sexual reproduction and produces oocysts (6, 7). Infected cats shed millions of unsporulated oocysts in their feces, which sporulate in the environment within 1–5 days, becoming infectious (4, 6). Cats also acquire infection by preying upon infected intermediate hosts, particularly rodents and birds, that harbor bradyzoite tissue cysts in their tissues (4, 5). In cats, the parasite completes its sexual cycle, typically presenting with mild, self-limiting gastrointestinal symptoms during acute infection (8–10); however, clinical disease occurs primarily in immunocompromised animals or those co-infected with feline leukemia virus or feline immunodeficiency virus, where affected cats may exhibit fever, anorexia, lethargy, pneumonia, hepatitis, and neurological signs (11).

Meat animals, including sheep, goats, pigs, cattle, and poultry, frequently suffer severe reproductive consequences—abortion, stillbirth, neonatal mortality, and congenital malformations—resulting in substantial economic losses and significant food safety concerns (12–16). Small ruminants are particularly susceptible, with ovine toxoplasmosis being a leading infectious cause of reproductive failure in sheep flocks globally (17–19). Among livestock, pigs, sheep, and goats demonstrate the highest rates of chronic infection (20–23), with clinical manifestations in susceptible animals including interstitial pneumonia, myocarditis, hepatic necrosis, and meningoencephalomyelitis, accompanied by fever, diarrhea, dyspnea, jaundice, and seizures (22).

Human infection occurs primarily through two routes: ingestion of oocysts from contaminated water, soil, vegetables, or fruits contaminated with cat feces, and consumption of undercooked meat containing tissue cysts from infected animals (24, 25). Vertical transplacental transmission from mother to fetus during primary infection poses significant risks for congenital toxoplasmosis (26, 27).

The parasite demonstrates extraordinary adaptability, transitioning between two distinct life stages that produce fundamentally different clinical outcomes: rapidly replicating tachyzoites responsible for acute infection, and slowly replicating bradyzoites enclosed within tissue cysts that establish lifelong chronic (latent) infection (28). All warm-blooded animals, including rodents, birds, mammals, and marine species, can serve as intermediate hosts harboring tissue cysts containing bradyzoites (5). Central to this success is the ability to invade any nucleated cell and establish a unique intracellular niche known as the parasitophorous vacuole (PV)—a membrane-bound compartment that provides a protected environment for replication while serving as a platform for host–parasite molecular interactions through the secretion of dense granule proteins (GRAs) (29, 30).

Following ingestion of oocysts or tissue cysts, sporozoites or bradyzoites convert to rapidly dividing tachyzoites that disseminate hematogenously to multiple organs. In immunocompetent humans, acute tachyzoite infection is usually subclinical and self-limiting, occasionally presenting as a mononucleosis-like illness with cervical lymphadenopathy, mild fever, and fatigue that resolves without treatment (31). However, severe acute disease can occur in immunocompetent individuals, particularly with certain virulent parasite strains, manifesting as ocular toxoplasmosis, pneumonitis, or hepatitis. In immunocompromised individuals — including those with advanced HIV/AIDS, transplant recipients, and patients receiving chemotherapy — primary acute infection or reactivation of latent infection can cause life-threatening toxoplasmic encephalitis, characterized by ring-enhancing brain lesions, altered mental status, and focal neurological deficits (32). Congenital toxoplasmosis arises from transplacental transmission of tachyzoites during primary maternal infection; while many congenitally infected neonates may initially appear asymptomatic, a subset manifests chorioretinitis (inflammation of the retina and choroid), intracranial calcifications, hydrocephalus, seizures, developmental delay, and hearing loss (33–36).

Following immune control of acute tachyzoite replication, the parasite converts to slowly dividing bradyzoites that form thick-walled tissue cysts, predominantly in the brain, retina, and skeletal muscle, establishing lifelong latent infection (28). In immunocompetent hosts, chronic bradyzoite infection is typically asymptomatic, as the immune system maintains cysts in a dormant state without eliminating them. However, ocular toxoplasmosis — the leading cause of infectious posterior uveitis worldwide — can result from either acute infection or reactivation of chronic bradyzoite cysts in the retina, characterized by necrotizing retinochoroiditis (inflammatory destruction of the retina and choroid) that can progressively destroy retinal tissue and cause irreversible vision loss (37–39). In immunocompromised patients, disruption of immune surveillance allows bradyzoite-to-tachyzoite reconversion, triggering reactivation disease most commonly presenting as toxoplasmic encephalitis (33, 40). The persistence of bradyzoite cysts in neural tissue also underlies emerging evidence linking chronic *T. gondii* infection to neuropsychiatric manifestations, including behavioral changes and potentially increased risk of certain psychiatric conditions, though causal relationships remain under investigation.

Dense granules are specialized secretory organelles that release their contents into the PV following parasite invasion (41). The proteins they deliver—collectively termed GRA proteins—perform diverse functions essential for parasite survival, including nutrient acquisition, immune modulation, and maintenance of the host–parasite interface (42–44). GRA proteins exhibit a wide molecular weight range from approximately 19 kDa to over 70 kDa, though not all GRA family members have been biochemically characterized (45). Many contain signal peptides directing their secretion into the PV lumen, the PVM, or beyond into the host cell cytoplasm and nucleus. Key effectors such as GRA15, GRA16, and GRA24 directly manipulate host cell signaling pathways, including activation of the Nuclear Factor kappa-light-chain-enhancer of activated B cells (NF-κB) pathway, stabilization of p53, and modulation of Mitogen-Activated Protein Kinase (MAPK) signaling, respectively (46–48). The intravacuolar network (IVN), a membranous tubular structure within the PV constructed by GRA2 and GRA6, facilitates nutrient uptake and waste removal (49, 50). Additionally, GRA proteins mediate host mitochondrial recruitment to the PVM via mitochondrial association factor 1 (MAF1) and other factors, thereby ensuring an adequate energy supply for replicating parasites (51, 52). The molecular mechanisms governing GRA protein trafficking, including translocation across the PVM via the Myb-related protein (MYR) translocon complex, represent sophisticated adaptations enabling the parasite to manipulate host cellular machinery while remaining sequestered within its protective vacuolar niche (53, 54).

Recent advances in molecular genetics, proteomics, and imaging have revealed the remarkable complexity and specificity of GRA-mediated pathogenesis, opening new avenues for therapeutic intervention and vaccine development (55, 56). Despite significant progress in characterizing individual GRA proteins, an integrated understanding of how these effectors orchestrate host–parasite interactions remains incomplete. Moreover, the translational potential of GRA proteins as diagnostic biomarkers, vaccine candidates, and therapeutic targets warrants systematic evaluation.

## 2 Role of GRA proteins in *Toxoplasma gondii* pathogenesis

**Figure 2:**
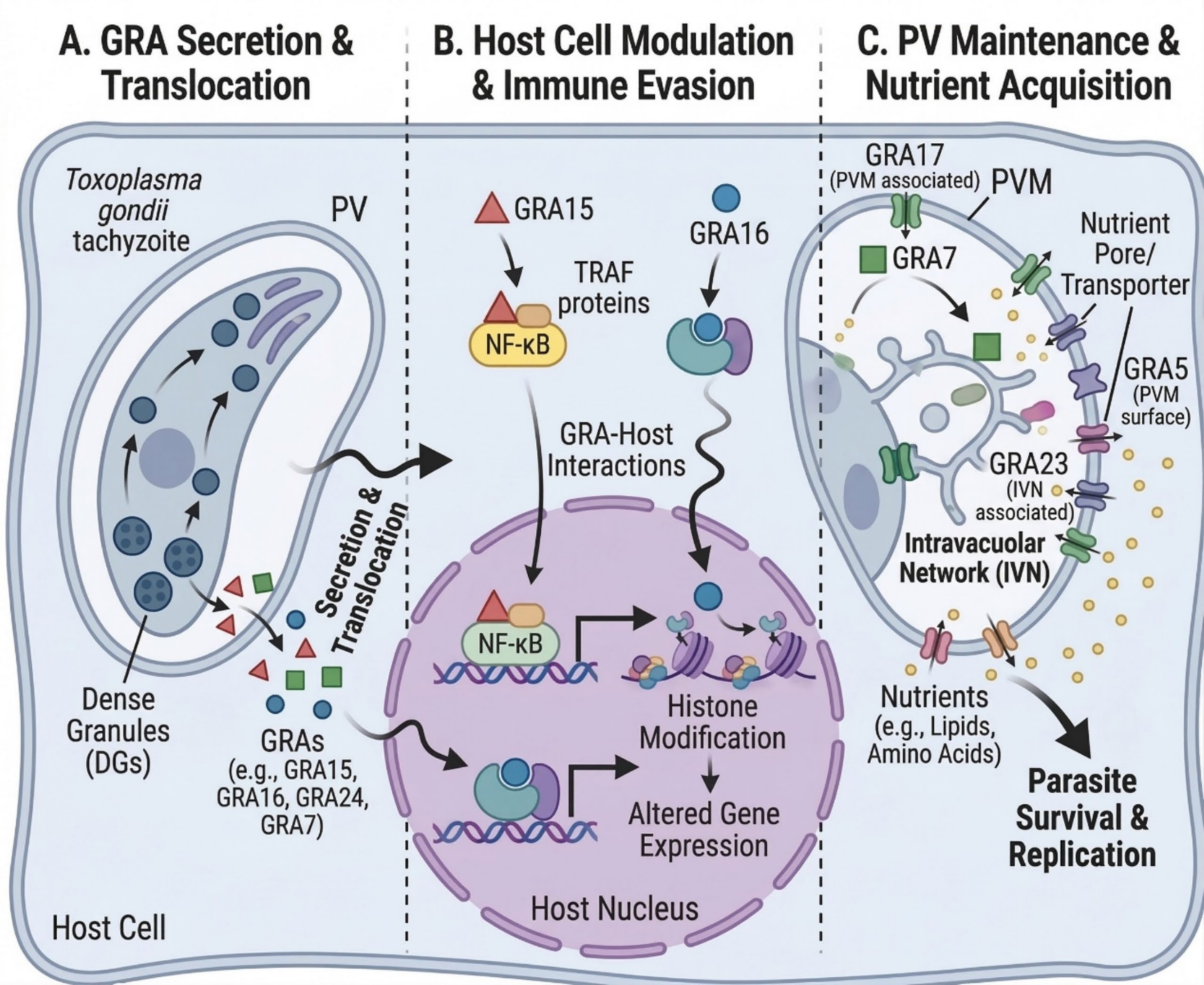
Dense granule proteins (GRAs) mediate *T. gondii* pathogenesis by host-manipulating cells and acquiring nutrients. **(A)** GRA secretion and translocation. Following host cell invasion, *T. gondii* tachyzoites release GRAs, including GRA15, GRA16, and GRA24, into the parasitophorous vacuole (PV). These GRAs are subsequently translocated across the PV membrane into the host cell cytoplasm. Notably, translocation of nuclear effectors (GRA16, GRA24) is MYR-dependent, while others, such as GRA15, may act primarily at the parasitophorous vacuole membrane (PVM). GRA7 localizes to the PVM. **(B)** Host cell modulation and immune evasion. Translocated GRAs manipulate host signaling and gene expression. GRA15 activates NF-κB signaling indirectly by interacting with TRAF adaptor proteins rather than by direct binding to NF-κB, while GRA16 traffics to the host nucleus to modulate gene expression. Within the nucleus, NF-κB–dependent transcription and GRA-mediated histone modifications alter host gene expression programs, suppressing immune responses and promoting parasite persistence. **(C)** PV maintenance and nutrient acquisition. GRA17 associates with the PVM to regulate nutrient pore/transporters, while GRA5 localizes to the PVM surface, maintaining the host–parasite interface. Within the PV lumen, GRA7 and GRA23 associate with the intravacuolar network (IVN), a membranous tubular structure that facilitates the uptake of host-derived nutrients, including lipids and amino acids, which are essential for parasite survival and replication. Created with BioRender.com.

The GRAs constitute a diverse family of secreted effectors that fundamentally shape *T. gondii* pathogenesis across all stages of infection. Following host cell invasion, GRAs are secreted into the PV and subsequently trafficked to multiple subcellular compartments, including the PVM, host cell cytoplasm, and host cell nucleus, where they orchestrate three interconnected processes essential for parasite survival: immune evasion, chronic persistence, and nutrient acquisition (Figure 2). The structural and metabolic roles of key GRA proteins are summarized in Table 1, while their immune modulation functions are detailed in Table 2. These proteins exhibit remarkable functional versatility—forming nutrient pores in the PVM, constructing the IVN for molecular trafficking, recruiting host organelles to fuel parasite metabolism, and manipulating host signaling pathways to subvert immune responses. The strategic deployment of GRAs enables *T. gondii* to establish a highly permissive intracellular niche during acute infection and subsequently transition to the encysted bradyzoite form, which is responsible for lifelong chronic infection. Understanding the molecular mechanisms by which GRAs mediate these processes not only illuminates fundamental aspects of host–parasite interactions but also identifies potential therapeutic targets for intervention against both active and latent toxoplasmosis.

**Table 1:** Structural and metabolic GRA proteins (PVM/IVN architecture and nutrient transport).

GRA Protein	Localization	Key functions	Phenotype of deletion	References
GRA1	PV lumen, IVN	IVN maintenance, nutrient salvage (glucose, pantothenate), secretion of other GRAs including GRA17, ATP production support	Growth arrest, severe metabolic defects, impaired ATP production, reduced glycolysis/TCA cycle	Zheng et al. (73)
GRA2	IVN, PVM	IVN architecture, lipid acquisition, cyst matrix organization, membrane trafficking	Altered IVN structure, growth defects, reduced chronic cyst burden	(82, 155)
GRA3	PV, PVM, host Golgi	Golgi interaction, host transport dysregulation, ER stress-induced apoptosis	Reduced virulence in some contexts, chronic infection defect	Fox et al. (82)
GRA7	PVM	NF-κB activation (partial), immune modulation, IVN uptake of host vesicles	Reduced NF-κB activation, altered virulence, chronic infection defect	Fox et al. (82)
GRA12	PVM, IVN membranes	IFN-*γ* resistance, chronic infection, cyst development, PV maintenance during IFN-*γ* attack, most critical virulence factor	Failed chronic infection, major virulence defect, avirulence, collapsed PV in IFN-*γ* conditions	(82, 130)
GRA12A-D	IVN, Cyst wall/matrix	Family of GRA12-related proteins, cyst formation, maintenance, and reactivation from chronic infection	Variable effects on cyst burden and reactivation efficiency	Guevara et al. (64)
GRA14	PVM	NF-κB activation (partial), ESCRT interaction, immune modulation, PVM maintenance	Reduced NF-κB activation, chronic infection defect	Fox et al. (82)
GRA17	PVM (pore component)	Small molecule permeability (up to 1.3–1.9 kDa), nutrient uptake, forms heptameric pore, essential for parasite survival	Aberrant PV morphology (‘bubble vacuole’), parasite death due to metabolic failure, avirulence in mice	(62, 69)
GRA23	PVM (pore component)	Small molecule permeability, nutrient uptake, works redundantly with GRA17, synthetically lethal with GRA17	Severe growth defect when combined with GRA17 deletion, synthetically lethal with GRA17	Gold et al. (62)
GRA38	PV (novel GRA)	Novel GRA identified via BirA* proximity labeling; secreted into parasitophorous vacuole; related to GRA39 and GRA40	No altered phenotype observed in Δgra38 parasites	Nadipuram et al. (91)
GRA39	PV, IVN	Lipid regulation critical for parasite growth; IVN lipid homeostasis; identified via BirA* proximity labeling; related to GRA38 and GRA40; key role in parasite replication and pathogenesis	Δgra39: slow-growing parasites, striking lipid deposits in parasitophorous vacuole, dramatically reduced virulence, lower tissue cyst burden in vivo	Nadipuram et al. (91)
GRA45	PV, Dense granules	Chaperone-like function (small heat shock protein domain), critical for correct localization of multiple GRAs to PVM, trafficking of GRA5, GRA23, termed ‘Blacksmith’ protein	Enhanced IFN-*γ* susceptibility, widespread GRA mislocalization, reduced virulence, synthetically lethal with GRA17, pleiotropic defects	Paredes-Santos et al. (69)
GRA47	PVM (pore component)	Small molecule permeability, forms heptameric pore with conserved histidines, interacts with GRA72	Aberrant ‘bubble vacuole’ morphology, reduced growth, reduced virulence	Bitew et al. (71)
GRA64	PV, IVN, PVM	ESCRT protein interaction (host endosomal sorting complex), vesicle formation in cysts, exposed to host cytoplasm	Enlarged vesicular structures in tissue cysts during chronic infection, ultrastructural abnormalities	Mayoral et al. (156)
GRA72	PVM (pore component)	Small molecule permeability, forms hexameric pore (distinct from GRA47), synthetically lethal with GRA17	Aberrant PV morphology, growth defects, reduced virulence	Bitew et al. (71)
GRA76	PV	Replication support, bradyzoite differentiation, virulence, stage conversion	Reduced virulence, lower cyst burden, vaccine potential	Zheng et al. (70)
GRA86	PV	Virulence factor, bradyzoite differentiation, stage conversion	Attenuated virulence, altered cyst formation	Zheng et al. (43)

**Table 2:** Immune modulation, host cell manipulation, and effector trafficking GRA proteins.

GRA protein	Localization	Key functions	Phenotype of deletion	References
GRA15	PVM, exported to host	Potent NF-κB activation via TRAF2/TRAF6 interaction; IL-12/IL-1β/TNF induction; Th1 response; GBP1 recruitment; strain-dependent polymorphism; Recombinant protein acts as a vaccine inducing humoral (IgG) and cellular (IFN-*γ*) immunity.	Reduced innate immunity, variable virulence effects depending on strain/host, lower IL-12 production, impaired Th1 response. Vaccination with rGRA15: Confers protective immunity (increased survival, reduced burden) against homologous type II strain	(46, 98, 114, 157)
GRA15	PVM, exported to host	Potent NF-κB activation via TRAF2/TRAF6 interaction; IL-12/IL-1β/TNF induction; Th1 response; GBP1 recruitment; strain-dependent polymorphism; Recombinant protein acts as a vaccine inducing humoral (IgG) and cellular (IFN-*γ*) immunity.	Reduced innate immunity, variable virulence, lower IL-12, impaired Th1 response. Vaccination with rGRA15: Confers protective immunity (increased survival, reduced burden) against homologous type II strain	Hasan et al. (157)
GRA16	Exported to host nucleus	Nuclear translocation, host transcription modulation, pp2a phosphatase interaction, immune evasion	Impaired intracellular growth, altered host gene expression	Franco et al. (126)
GRA24	PVM, exported to host nucleus	p38 MAPK activation, innate cytokine induction (IL-12, IL-1β), synergizes with GRA15 for maximal immune response, nuclear translocation	Reduced innate immunity (synergizes with GRA15), decreased IL-12/IL-1β production, attenuated p38 activation	(47, 114)
GRA28	Host nucleus	Chromatin accessibility modulation, boosts NF-κB-dependent transcription at CCR7 locus, promotes macrophage hypermigration for dissemination	Reduced macrophage chemotaxis, impaired parasite dissemination in vivo	(47, 101)
GRA29	PV lumen, Cyst matrix	Forms filamentous strands in the PV lumen; redistribution throughout the cyst matrix during bradyzoite differentiation; strand formation regulated by C-terminal phosphorylation.	Non-essential for viability; disrupts intravacuolar filament architecture (strands); potential undiscovered role in cyst biology.	Young et al. (158)
GRA35	PV, PVM	Inflammasome activation (NLRP1 in rat macrophages), pyroptosis induction, immune recognition, lipid regulation	Reduced pyroptosis in rat macrophages, fitness defect	(67, 142)
GRA36 and GRA54	PV	Unknown specific function, serodiagnostic antigen candidate (poor performance)	Unknown	Sołowińska and Holec-Gąsior (142)
GRA39	PV, IVN (Vaccine candidate)	DNA vaccine (pVAX-TgGRA39) induces humoral immunity (IgG, IgG2a) and cellular immunity (Th1, Th17, Tc17); increases IFN-*γ*, IL-2, IL-12, IL-17A, IL-17F, IL-22, IL-23; elevates CD3 + CD4 + and CD3 + CD8 + T cells; upregulates ROR*γ*t, RORα, STAT3, IL-6, TGF-β1, IL-1β for Th17/Tc17 activation.	DNA immunization confers protection: prolonged survival (16.80 ± 3.50 days) against virulent RH strain (Type I); 44.5% reduction in brain cysts against PRU strain (Type II). No significant IL-4/IL-10 change (Th2 response minimal).	Zhu et al. (159)
GRA42 and GRA43	PV, PVM	GRA localization regulation (chaperone for GRA17, GRA23, GRA35), inflammasome activation in rat macrophages, quality control for effector trafficking	Reduced pyroptosis, fitness defect, GRA mislocalization (GRA17/23/35 fail to reach PVM)	Wang et al. (67)
GRA44	PV, PVM	Effector export (GRA16, GRA24, GRA28) across PVM into host cell, works with GRA45 and MYR1 translocon complex, essential for host nuclear effector delivery	Complete block in GRA16 export, impaired growth, loss of host nuclear effector delivery	(126, 127, 160)
GRA83	PV	Stimulates innate immunity via NF-κB activation (p65 nuclear translocation); induces IL-12 and IFN-*γ* production; functions in a pathway convergent with GRA15; limits acute and chronic parasite burden.	Increased virulence during acute infection; higher parasitemia and cyst burden; reduced IL-12/IFN-*γ* production; increased tissue inflammation; no additive effect with GRA15 deletion.	Thind et al. (120)

## 3 Nutrient acquisition strategies

### 3.1 PVM permeability and pore formation

#### 3.1.1 Pore-forming GRAs in acute tachyzoite infection

*T. gondii* establishes a specialized intracellular compartment—the parasitophorous vacuole—that serves as its replication niche (57, 58). The PVM, initially derived from the host plasma membrane, functions as both a protective barrier against host immune mechanisms and an interface for nutrient exchange (59). Since classical transporters have not been identified in this membrane (60), nutrient pores formed by GRA17 and GRA23 modulate selective permeability (Figure 2), allowing the passage of molecules up to 1,300–1,900 Da while mediating the excretion of metabolic by-products (61, 62).

#### 3.1.2 Pore-forming GRAs in chronic bradyzoite cysts

The functional importance of GRA17 extends beyond the tachyzoite stage. During conversion to encysted bradyzoites, the PVM transitions to become the cyst membrane, forming the outer layer of the cyst wall (63). This transition has been confirmed through tracking PVM-associated GRA5 and GRA7 (Figure 2) during *in vitro* differentiation, with both proteins visible at the cyst periphery as early as 6 h post-differentiation (64). Furthermore, Δgra3, Δgra5, Δgra7, Δgra8, and Δgra14 mutants revealed that PVM-localized GRAs are crucial for normal accumulation of cyst wall proteins, indicating their essential role in cyst wall and cyst membrane development (64).

The clinical significance of these findings is substantial, as *T. gondii* establishes chronic infection by forming thick-walled tissue cysts that persist in the brain (65). Cysts persisting in the presence of perforin-mediated CD8 + T-cell immunity exhibit significantly elevated mRNA levels for GRA1, GRA2, GRA3, GRA7, and ROP35. Notably, GRA3-deficient parasites were significantly more efficiently eliminated by anti-cyst CD8 + T cells, indicating that GRA3 mediates cyst persistence through resistance or evasion of T-cell immunity (65). Consistent with these observations, deletion of GRA17 in the type II ME49 strain resulted in reduced parasite growth and formation of grossly enlarged bubble vacuoles with diminished PVM permeability (66). Although ME49 Δgra17 parasites formed cysts *in vitro* at rates comparable to those of wild-type parasites, bradyzoite viability was significantly reduced. Complementation with the bradyzoite-specific SRS9 promoter dramatically increased viability, underscoring GRA17’s critical role in regulating bradyzoite survival (66). Moreover, mice infected with high doses of ME49 Δgra17 parasites showed no brain parasites, indicating that GRA17 plays essential roles during both acute tachyzoite infections and chronic bradyzoite persistence.

Genome-wide screens have identified additional proteins involved in PVM nutrient acquisition and trafficking. GRA57, GRA70, GRA71, and GRA72 are required for correct localization of GRA17/GRA23 to the PVM, while GRA42, GRA43, and the chaperone-like GRA45 also participate in this process (67–69). GRA47 and GRA72 have been identified as additional contributors to PVM permeability; GRA72 is synthetically lethal with GRA17, and its deletion produces bubble vacuoles with reduced permeability similar to GRA17 knockouts (69). Structural modeling using AlphaFold2-multimer predicts that GRA72 forms a hexameric pore structure (69), and GRA47 interacts with GRA72, with the loss of either protein impairing PV morphology, small-molecule transport, growth, and virulence (70, 71). Importantly, Pitman et al. demonstrated that the *Plasmodium falciparum* ortholog PfEXP2 can restore PV permeability defects in GRA17/GRA23 mutants, whereas overexpression of individual GRA proteins provided only partial rescue, indicating that these proteins regulate nutrient acquisition through distinct, non-redundant mechanisms (72).

Proper localization of pore-forming proteins depends on GRA1, which maintains the structural integrity of the IVN—membranous tubules mediating molecular transport within the PV (73). GRA1 depletion disrupts IVN architecture, impairs secretion of GRA proteins, including GRA17, to the PVM, and severely reduces nutrient uptake, including glucose and pantothenate. Consequently, GRA1-deficient parasites exhibit metabolic defects including reduced glycolysis and TCA cycle activity, decreased ATP levels, organelle damage, slower growth, and increased bradyzoite differentiation (73).

Beyond passive pore-mediated diffusion, *T. gondii* actively scavenges host proteins via an ingestion pathway initiated by GRA14, which recruits host endosomal sorting complex required for transport (ESCRT) machinery to bud vesicles into the PV (74). These vesicles are trafficked to the plant vacuole-like compartment (PLVAC), where cathepsin protease L (CPL) degrades cargo and the chloroquine resistance transporter (CRT) exports peptides to the parasite cytosol. Ingestion-deficient mutants exhibit increased reliance on biosynthetic pathways and more severe growth defects under amino acid limitation, confirming this pathway’s role in nutrient scavenging (74). Complementing this mechanism, GRA64, a transmembrane protein localized to the PVM, interacts with host ESCRT components. Although Δgra64 does not affect acute virulence or encystation, ultrastructural analysis revealed enlarged vesicular structures underneath the cyst membrane, suggesting a role in organizing ESCRT recruitment and subsequent intracystic vesicle formation (75). Together, these findings highlight the versatility of nutrient acquisition in *T. gondii*—combining IVN-dependent protein trafficking, passive pore-mediated uptake, and active protein ingestion—which underlies the parasite’s ability to thrive across diverse hosts during both acute and chronic infection stages.

#### 3.1.3 Intravacuolar network and nutrient salvage

The IVN is a highly specialized, membranous, tubular structure that extends throughout the PV lumen and is one of the most distinctive features of *T. gondii* infection. The IVN forms shortly after GRA protein secretion, establishing a network of highly curved membrane tubules that are hypothesized to facilitate PV expansion and maintenance by increasing surface area for nutrient exchange (50, 75). GRA1 plays a critical role in maintaining IVN structural integrity; its inactivation alters membranous tubule structures that mediate molecular transport, impairs secretion of various GRA proteins, including GRA17, to the PVM, and causes severe defects in nutrient absorption, including glucose and pantothenate. Consequently, mutants lacking GRA1 exhibit slower growth and a greater propensity to differentiate into bradyzoites (73).

IVN biogenesis is primarily driven by GRA2 and GRA6. Mercier et al. (76) demonstrated that the unique nanotubule conformation is induced by GRA2 and further stabilized by GRA6; GRA2 induces formation of curved tubules from vesicular material secreted from the posterior end of the parasite, while GRA6 stabilizes the curvature (75, 76). Vacuolar compartments generated by GRA2 knockout parasites are dramatically disorganized, with the normally tubular network replaced by small, aggregated material, and complementation studies revealed that both amphipathic *α*-helices are required for correct network formation and membrane tubulation (76, 77). Similarly, Δgra6 mutants display altered mature networks, characterized by small vesicles rather than elongated nanotubules (76).

Multiple additional GRA proteins associate with IVN membranes: GRA2, GRA4, GRA6, and GRA9 all traffic exclusively to the IVN, with GRA4 and GRA6 likely anchored via transmembrane domains, whereas GRA2 and GRA9 contain hydrophobic α-helices facilitating membrane interaction (50). Cross-linking studies have demonstrated that GRA4 and GRA6 specifically interact with GRA2 to form a multimeric complex stably associated with the IVN, which may participate in nutrient or protein transport within the vacuole (78). GRA12 presents an interesting case: although implicated in processes related to the establishment (79), subsequent studies have demonstrated normal IVN ultrastructure in Δgra12 mutants, suggesting that GRA12 is not required for IVN biogenesis (80).

The PVM presents a selective barrier for nutrient exchange, and specific GRA proteins form pores to facilitate small-molecule transport. GRA17 and GRA23 are *α*-helical proteins homologous to the *Plasmodium* translocon protein EXP2, which form nutrient pores that allow the passage of molecules with molecular weights of 1,300–1,900 Da (62). ME49 Δgra17 parasites exhibit reduced growth and form grossly enlarged bubble vacuoles with reduced PVM permeability (66). Recent research identified additional pore-forming proteins: deletion of GRA47 results in aberrant bubble vacuole morphology and reduced small-molecule permeability, mirroring phenotypes observed in GRA17 and GRA72 knockouts. Structural predictions indicate that GRA47 and GRA72 form heptameric and hexameric pores, respectively, with conserved histidine residues lining the pore (71).

The IVN also serves as a critical platform for lipid acquisition, as the host provides a continuous supply of lipids to support PVM and IVN growth and maturation, with IVN membranes playing important roles in lipid salvage and in acquiring host cytosolic proteins (64, 81). Importantly, IVN-associated GRA proteins are essential for chronic infection establishment: deletion of PVM-associated GRA3, GRA7, GRA8, and GRA14, or IVN membrane-associated GRA2, GRA9, and GRA12 in the low-virulence type II Prugniaud strain, induces severe defects in chronic-stage cyst development *in vivo* without affecting parasite growth rate or ability to differentiate into cysts *in vitro* (82). These findings are further discussed in Section 4.4 in the context of tissue cyst formation and bradyzoite differentiation.

#### 3.1.4 Host organelle interactions

*Toxoplasma gondii* has evolved sophisticated mechanisms to recruit and manipulate host organelles, whereby the PVM forms intimate associations with host mitochondria and endoplasmic reticulum (ER). At the same time, the parasite actively subverts Golgi-derived vesicular trafficking pathways. Several GRA proteins mediate ER interactions: GRA3, GRA5, and GRA6 bind to calcium-modulating ligand (CAMLG) in the intracellular integral membrane of ER, suggested as the ligand for ER anchorage to the PVM during parasitism (83). Additionally, TgVIP1 mediates ER-PV interactions through host vesicle-associated membrane protein-associated proteins A and B (VAPA and VAPB), which localize at the PVM together with motile sperm domain-containing protein 2 (MOSPD2); cells deficient in VAPA, VAPB, and MOSPD2 do not recruit host ER at the PV, resulting in parasite growth defects (29). Another study demonstrated that TgROP1 establishes membrane contact sites (MCS) with host ER tethering proteins, revealing that Toxoplasma uses effectors from distinct secretory organelles to establish organelle-specific MCS: TgROP1 from rhoptries tethers host ER, whereas TgMAF1 from dense granules tethers host mitochondria (29).

GRA3 directly interacts with host Golgi membranes and disrupts vesicular transport by modulating host anterograde transport through binding to the Golgi apparatus (84). GRA3 associates with Golgi purified from CHO cells and binds phosphatidylinositol lipids via its C-terminus; GRA3 knockouts display reduced entry of host Golgi vesicles into the PV, supporting a role in lipid scavenging (41). The parasite exploits Golgi-derived vesicles for sphingolipid acquisition by subverting host Golgi structure, resulting in its fragmentation into numerous ministacks surrounding the PV, and hijacking vesicles marked with Rab14, Rab30, or Rab43 that colocalize with host-derived sphingolipids in the vacuolar space (85). Notably, GRA3 has also been implicated in pathological processes during chronic infection, inducing neural cell apoptosis via ER stress signaling via activation of the PERK-ATF4-CHOP pathway, which may contribute to toxoplasmic encephalitis (86). Strain-specific differences exist, with GRA3 gene expression being higher in less virulent strains, suggesting that elevated GRA3 expression might contribute to lower virulence by inducing neuronal apoptosis (86).

The Mitochondrial Association Factor 1 (MAF1) directly recruits host mitochondria to the PV. Host mitochondrial association (HMA) is present in type I and III strains but absent in type II strains; expression of MAF1 in type II tachyzoites confers a dramatic increase in HMA, with recruited mitochondria specifically associated with PVM portions that stain with anti-MAF1 (51). Loss and gain of HMA are associated with alterations in host cell immune gene transcription and the *in vivo* cytokine response during murine infection (51). Mechanistically, MAF1b binds to the multifunctional MIB protein complex on host mitochondria, and reduction of proteins in this complex reduces the close association between host mitochondria and the parasite’s vacuole (87), while mitochondrial receptor protein TOM70 and mitochondria-specific chaperone HSPA9 have been identified as essential HMA mediators (88).

GRA7 plays a crucial role in the formation of specialized structures for nutrient acquisition. *T. gondii* scavenges endolysosomes and lipid droplets containing cholesterol from host cells through host organelle-sequestering tubulostructures (HOST), with GRA7 associating with the cytosolic face of host membranes to stabilize these structures. GRA7 forms the dense coat distributed on the HOST surface (89, 90). These lipid-filled vesicles can be internalized into the IVN. Although *T. gondii* encodes an LCAT-like protein (TgLCAT), the parasite lacks bona fide lecithin-cholesterol acyltransferase activity and is unable to esterify cholesterol *de novo*; the precise mechanism of vesicle internalization remains under investigation. Similarly, GRA39, identified through *in vivo* biotinylation (BioID) approaches, has been implicated in lipid regulation; Δgra39 parasites contain striking lipid deposits in the parasitophorous vacuole, display dramatically reduced virulence, and have lower tissue cyst burden in vivo (91).

The parasite also usurps host cellular machinery to sequester organelles via the ESCRT pathway. GRA14 and GRA64 at the PV bind to ALIX, which interacts with CHMP4B subunits that cluster to form deep membrane concavities; IVN tubules fuse with CHMP4B-induced tubules, contributing to their elongation and membrane supply (92). Bioinformatics analysis has identified TSG101- and ALIX-targeting motifs in GRA14 exposed to the host cytosol, facilitating these host–parasite interactions (92, 93). Notably, recruitment of different host organelles appears temporally regulated and potentially competitive: host ER is observed most readily early after infection, and host ER-*Toxoplasma* MCS limit the extent of host mitochondria-*Toxoplasma* MCS and vice versa (94). This competition may reflect shifting metabolic needs across different infection stages, highlighting the dynamic, coordinated nature of host organelle manipulation by *T. gondii*.

### 3.2 Immune modulation and host signaling

#### 3.2.1 GRA-mediated activation of host immune signaling pathways

**Figure 3:**
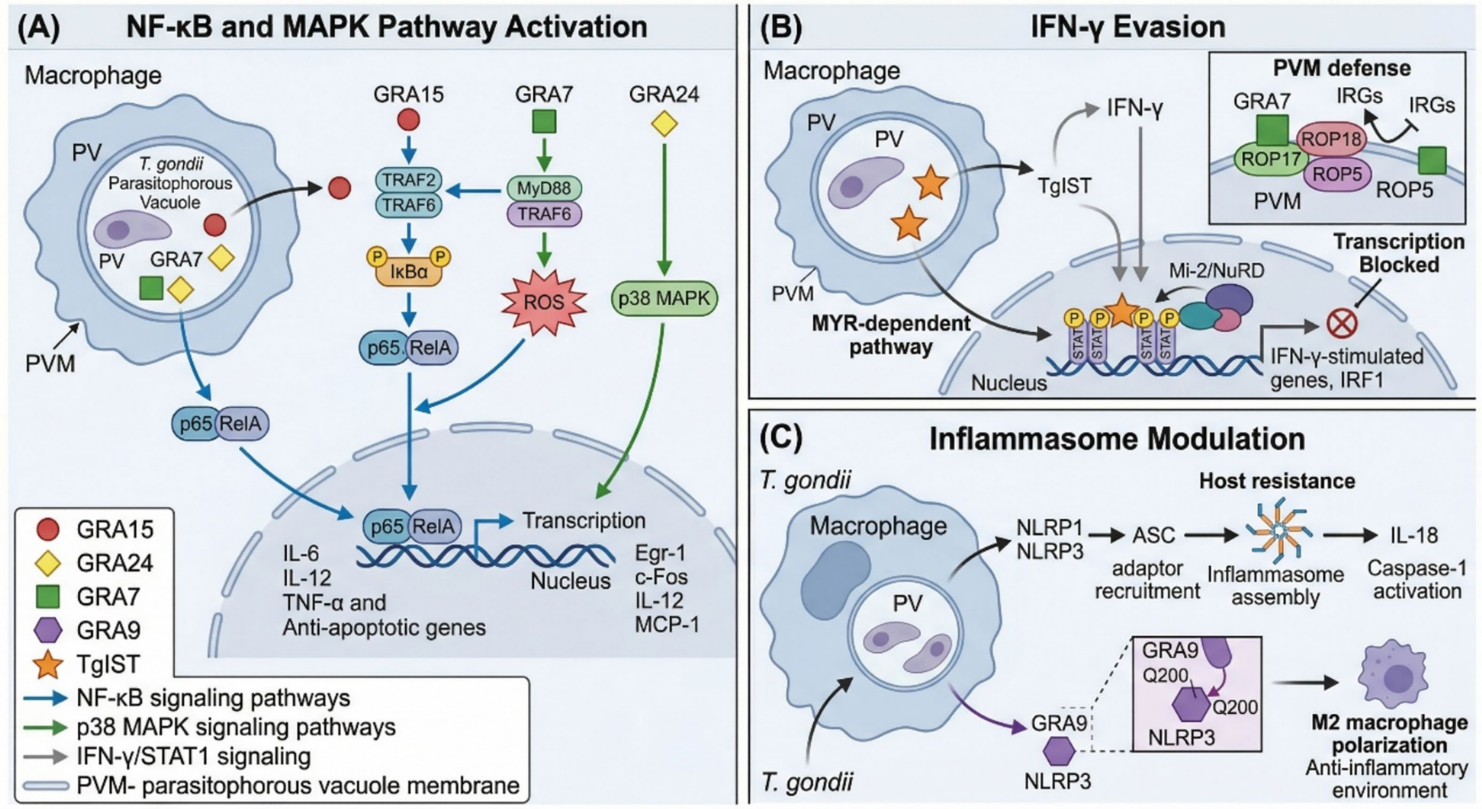
Role of GRA proteins in host immune signaling and evasion. **(A)** NF-κB and MAPK pathway activation. GRA15 activates NF-κB signaling via TRAF2/TRAF6 adaptor proteins, inducing IκB*α* phosphorylation and p65/RelA nuclear translocation, which drives transcription of pro-inflammatory cytokines (IL-6, IL-12, TNF-α) and anti-apoptotic genes. GRA7 activates the MyD88/TRAF6 axis, triggering ROS production and reinforcing NF-κB and MAPK signaling. GRA24 independently activates p38 MAPK, driving sustained nuclear translocation and upregulation of Egr-1, c-Fos, IL-12, and MCP-1. **(B)** IFN-*γ* evasion. TgIST is translocated to the host nucleus via the MYR-dependent pathway, where it sequesters phosphorylated STAT1 dimers on chromatin and recruits the Mi-2/NuRD repressive complex, blocking transcription of IFN-*γ*-stimulated genes, including IRF1. GRA7, in concert with ROP18, ROP17, and ROP5, resists IRG-mediated destruction of the PVM. **(C)** Inflammasome modulation. *Toxoplasma gondii* infection activates NLRP1 and NLRP3 inflammasomes, driving IL-18 production essential for host resistance. GRA9 counteracts this by binding NLRP3 via its C-terminal domain (residue Q200), blocking ASC adaptor recruitment and inflammasome assembly, and promoting a shift toward anti-inflammatory M2 macrophage polarization. Created with BioRender.com.

Multiple GRA proteins function as immune modulators by activating key host signaling pathways. The central mediators of this process are GRA15, GRA24, and GRA7, which collectively regulate NF-κB, MAPK, and inflammasome signaling (Figure 3).

GRA15 activates NF-κB through interaction with TRAF adaptor proteins rather than through direct NF-κB binding. Specific TRAF-binding sites on GRA15 are essential for NF-κB activation, with TRAF2 playing a critical role, as cells lacking TRAF2 display impaired GRA15-mediated NF-κB activation (46). The *T. gondii* HB1 strain’s unique GRA15 protein activates NF-κB by inducing IκBα phosphorylation and p65/RelA nuclear translocation, with the activation domain mapped to amino acids 194–518 (45, 95). This activation leads to upregulation of anti-apoptotic genes, including IAPs and Bcl-2 family members, and GRA15 also contributes to IFN-*γ*-mediated growth inhibition by recruiting the TRAF ubiquitin ligase to the PVM, demonstrating the complex balance between immune activation and evasion (96).

Using an NF-κB luciferase reporter assay, GRA7, GRA14, and GRA15 have been identified as pathway activators, with GRA15 being the most essential. Analysis of genetic knockout parasites (PruΔgra7, Δgra14, Δgra15) demonstrated that deletion of any of these GRAs impaired key NF-κB signaling steps: reduced IκBα phosphorylation, blocked RelA nuclear translocation, and decreased IL-6 production. RNA-seq analysis confirmed global downregulation of NF-κB-mediated gene expression, while mouse virulence assays revealed that all mutant strains showed increased virulence, proving that GRA-induced NF-κB activation is critical for limiting parasite expansion (97). GRA15 has also been identified as a key factor that induces seizures during chronic brain infection by activating the NF-κB pathway, thereby increasing expression of pro-inflammatory molecules, including IL-1β. Critically, inhibiting IL-1 receptor signaling reduced seizure severity in infected mice (98).

GRA7 triggers proinflammatory responses in macrophages by activating the MyD88/TRAF6 signaling pathway; GRA7 binds MyD88, thereby activating TRAF6 and inducing a reactive oxygen species (ROS) burst, which together induce NF-κB and MAPK signaling (99). The C-terminal region of GRA7 (GRA7-V) is both necessary and sufficient for interacting with and ubiquitinating TRAF6, and immunization with GRA7-V provided protective immunity in mice (99). GRA15 and GRA24 cooperatively reprogram infected macrophages into migratory cells, driving expression of the chemotactic receptor CCR7, while GRA28 further enhances this by boosting chromatin accessibility at the Ccr7 gene locus. *In vivo*, this cooperative induction of a pro-migratory state enables infected macrophages to efficiently migrate to secondary organs (100).

GRA24 activates p38 MAPK, which functions downstream of various receptors and can signal through MyD88-independent immune pathways, triggering sustained p38 MAPK phosphorylation in host macrophages that drives IL-12 production and protective Th1 immune responses (101). Mechanistically, GRA24 modulates host signaling by directly inducing prolonged p38α MAP kinase activation and nuclear translocation, leading to upregulation of the transcription factors Egr-1 and c-Fos and the production of proinflammatory cytokines, including IL-12 and MCP-1 (102). This persistent activation of host p38 MAPK signaling increases TIMP1 production, facilitating parasite migration across cellular barriers, including the intestinal wall and the blood–brain barrier, without causing major tissue disruption (103).

*Toxoplasma gondii* infection can trigger inflammasome activation and pyroptotic cell death in certain host cell types, particularly in rat macrophages (104, 105). Infection activates host NLRP1 and NLRP3 inflammasomes, which are critical for innate resistance; mice deficient in NLRP1, NLRP3, ASC, caspase-1/11, or the IL-18 receptor display decreased circulating IL-18, increased parasite replication, and acute mortality, establishing that inflammasome-driven IL-18 production is essential for controlling infection (106). Conversely, GRA9 actively modulates host inflammation by directly interacting with the NLRP3 inflammasome. Through its C-terminal domain and specifically the key residue Q200, GRA9 binds NLRP3 and blocks its association with the adaptor protein ASC, thereby inhibiting inflammasome assembly and promoting a shift from pro-inflammatory M1 to anti-inflammatory M2 macrophages (107). In murine sepsis models, the recombinant C-terminal protein demonstrated therapeutic potential by improving survival through dampened inflammation (107).

### 3.3 Immune evasion and chronic infection

#### 3.3.1 IFN-*γ* resistance

Interferon-gamma (IFN-*γ*) is a cornerstone of host defence against intracellular pathogens, activating cell-autonomous immune mechanisms to restrict parasite replication (68, 108, 109). GRA proteins employ multiple strategies to resist IFN-*γ*-mediated clearance, ranging from direct inhibition of STAT1 signaling to interference with immunity-related GTPases (IRGs) (Figure 3). Control of cerebral infection critically depends on IFN-*γ* produced by T cells, which activates cerebral immune cells to suppress tachyzoite proliferation and recruits T cells to infection sites; CD8 + T cells can also directly eliminate chronic cysts via perforin (102).

Among IFN-*γ* resistance effectors, Toxoplasma Inhibitor of STAT1 Transcription (TgIST), which is commonly classified as a GRA protein, traffics to the host cell nucleus and blocks IFN-*γ* signaling. This effector translocates to the host cell nucleus, where it inhibits STAT1-dependent proinflammatory gene expression through a dual mechanism: sequestering activated STAT1 on chromatin and simultaneously recruiting the nucleosome-remodelling deacetylase (Mi-2/NuRD) complex to create a transcriptionally repressive chromatin environment (109–111). TgIST also inhibits STAT1/STAT2 heterodimer-mediated transcription following type I IFN stimulation, globally suppressing IFN-stimulated gene expression (112). In the context of host cell death suppression, TgIST-mediated STAT1 inhibition reduces the expression of pro-apoptotic IFN-stimulated genes, thereby contributing to host cell survival during infection. Structural studies have revealed that TgIST contains a repeat region that binds specifically to phosphorylated STAT1 dimers, displaces the transcriptional co-activators CBP/p300, and blocks gene expression (113). TgIST-deficient parasites are rapidly eliminated by host inflammatory monocytes during acute infection, highlighting their essential role in resisting IFN-*γ*-mediated killing (109, 112).

The transition from tachyzoite to bradyzoite represents a fundamental shift in GRA functional priorities. During the tachyzoite stage, GRA15 functions primarily as a potent immune activator driving NF-κB-dependent proinflammatory responses. As parasites differentiate into bradyzoites, however, the role of GRA15 is recalibrated to support chronic persistence rather than acute immune engagement. In bradyzoites, the MYR1-dependent effector export system remains functional, and stage-specific regulation of GRA15 expression contributes to the dampened inflammatory profile characteristic of chronic cysts, minimizing immune detection while maintaining essential host–parasite interactions. GRA15 polymorphisms across T. gondii lineages further modulate this balance, with strain-specific differences in GRA15 contributing to differential virulence and immune responses that influence the outcome of both acute and chronic infection (96). In human macrophages, GRA15 and GRA24 synergistically induce the secretion of IL-12, IL-18, and IL-1β, which subsequently activate IFN-*γ* production by peripheral blood mononuclear cells, thereby controlling Toxoplasma proliferation (47, 96, 114). Paradoxically, GRA15 can also suppress IFN-*γ*-induced IDO1-dependent antiparasitic responses in human cells by inducing IL-1β secretion from monocytes, which stimulates iNOS expression and nitric oxide production that inhibits IDO1 activity (115). This context-dependent duality — promoting protective immunity during acute infection while modulating immune detection during chronic persistence — reflects the evolutionary pressure on T. gondii to balance clearance resistance with long-term host survival.

GRA7 functions in concert with rhoptry proteins ROP18, ROP17, and ROP5 to resist IFN-*γ*-activated immunity-related GTPases (IRGs), which target the parasitophorous vacuole for destruction in murine cells (116, 117). GRA12 has been identified as a major virulence factor required for parasite resistance to host IFN-*γ*; both type I and type II strain Δgra12 parasites ultimately succumb to IFN-*γ*-activated host cell innate immunity, despite initially resisting IRG coating of the PVM (82). Additionally, GRA6 stimulates microglial IFN-*γ* production via its N-terminal region, potentially serving as a sentinel system to detect cerebral parasite proliferation (118).

Genome-wide CRISPR screens have identified 353 *Toxoplasma* genes that determine parasite fitness in IFN-*γ*-activated macrophages, including GRA45, which functions as a chaperone-like protein critical for correct GRA localization and secretion (68). From a translational perspective, GRA proteins—particularly GRA4, GRA6, and GRA7—have demonstrated high immunogenicity and are considered promising vaccine candidates (119). The dual nature of certain GRA proteins, simultaneously activating protective immunity while providing immune evasion mechanisms, highlights the evolutionary pressure on *Toxoplasma* to balance virulence with chronic persistence (108, 120).

#### 3.3.2 Suppression of host cell death pathways

*Toxoplasma gondii* has evolved sophisticated mechanisms to manipulate host cell death pathways through secretion of GRAs and rhoptry proteins (ROPs) targeting multiple signaling cascades (121, 122). Parasite survival critically depends on preventing premature host cell death, as apoptosis, necroptosis, and pyroptosis would terminate the intracellular replicative niche (123). Central to this anti-apoptotic strategy is the inhibition of caspases-3, −8, and −9, thereby shutting down both intrinsic and extrinsic apoptotic pathways, mediated by the secretion of inhibitory apoptotic proteins (IAPs) and by modulation of Bcl-2 family proteins. It should be noted that direct experimental evidence linking specific GRA proteins to caspase-12 inhibition has not been conclusively demonstrated (123). GRA3, GRA5, and GRA6 have been shown to bind calcium-modulating ligand (CAMLG), thereby potentially regulating calcium signaling. However, direct experimental evidence linking these specific GRA proteins to caspase inhibition or to modulation of apoptosis remains limited, and the proposed anti-apoptotic roles require further experimental validation (124, 125).

GRA16 is another critical nuclear-targeted effector that modulates host cell survival. This protein is exported to the host nucleus via the MYR1-dependent pathway, where it binds to host ubiquitin-specific protease HAUSP and phosphatase PP2A-B55 (48). These interactions modulate p53 activity and cell cycle progression, promoting host cell survival. The MYR1 translocation machinery, comprising MYR1, MYR2, MYR3, MYR4, GRA44, GRA45, ROP17, and ASP5, is essential for the export of numerous effectors across the PVM (126–128).

The effector HCE1/TEEGR modulates the host cell cycle by binding to E2F transcription factors, thereby upregulating cyclin E and promoting S-phase entry, thereby contributing to host cell survival (129). GRA12 has been identified as a strain- and host-species-transcendent virulence factor that protects parasites from IFN-*γ*- activated macrophage clearance by preventing PVM collapse and host-cell necrosis (130). Importantly, bradyzoites maintain the ability to suppress host cell death during chronic infection; the MYR1-dependent protein export system remains functional, and TgIST continues to limit IFN-*γ* signaling (131). This protection is critical for the long-term persistence of tissue cysts.

### 3.4 GRA proteins in tissue cyst formation and bradyzoite differentiation

The transition from rapidly dividing tachyzoites to slowly replicating bradyzoites contained within tissue cysts is essential for *T. gondii’s* chronic infection and transmission (28, 132). This differentiation involves extensive remodeling of the parasitophorous vacuole into a heavily glycosylated cyst wall, with GRA proteins playing fundamental roles throughout this process.

Studies tracking PVM-associated GRA proteins during cyst development demonstrated that GRA5 and GRA7 localize to the cyst membrane and cyst wall region throughout differentiation, supporting the model that the PVM transitions directly into the cyst membrane (64). By day 3 post-differentiation, these proteins appear in a continuous pattern at the cyst periphery, indicating the establishment of mature cyst structure. Genetic deletion studies revealed that Δgra3, Δgra5, Δgra7, Δgra8, and Δgra14 mutants all exhibit reduced accumulation of cyst wall cargo proteins at the cyst periphery, demonstrating that PVM-associated GRAs are crucial for proper cyst wall maturation (64). The IVN also plays dynamic roles during cyst development; GRA2 and GRA12, which are associated with the IVN, contribute to cyst matrix and wall formation and regulate the temporal and spatial organization of proteins during the tachyzoite-to-bradyzoite transition (75).

Proximity biotinylation has enabled the identification of novel bradyzoite-specific GRAs. Using a MAG1-BirA* fusion as bait (133), identified several bradyzoite-upregulated GRAs, including GRA50-53 and GRA55. While GRA55 is dispensable for *in vitro* growth and acute virulence, its deletion results in approximately a 10-fold reduction in brain cysts during chronic infection, with complementation rescuing this phenotype. Additional novel GRAs (GRA85-88) localize to the parasitophorous vacuole in tachyzoites and to the cyst matrix in bradyzoites, colocalizing with GRA12 (43). GRA86 is particularly important for virulence and brain cyst formation; deletion in type II Pru parasites results in significantly reduced virulence and fewer brain cysts, although *in vitro* growth remains unaffected.

Contrary to previous assumptions of bradyzoite dormancy, recent studies have revealed that bradyzoites within tissue cysts are dynamic, replicating entities. Novel imaging approaches using TgIMC3 labeling have demonstrated not only sporadic and asynchronous division but also synchronous replication of all bradyzoites within mature tissue cysts (134, 135). This evidence of cyclical, episodic bradyzoite growth has significant implications for understanding the dynamics of chronic infection. Human myotube-based *in vitro* systems can generate orally infectious cysts that tolerate antibiotic exposure and temperature stresses (136). Metabolomic characterization reveals global metabolic changes, including elevated amino acid levels and decreased levels of nucleobase- and TCA cycle-associated metabolites, reflecting distinct bradyzoite metabolic requirements.

GRA8 and GRA9 also contribute to cyst biology through association with bradyzoite pseudokinase 1 (BPK1), a cyst wall component crucial for oral infectivity. BPK1 forms complexes with GRA8, GRA9, and MAG1, and its deletion results in cysts that are more sensitive to pepsin-acid treatment and have reduced oral infectivity, indicating a role in cyst wall integrity (137). In summary, GRA proteins orchestrate a complex program that ensures host cell survival during acute infection while enabling the developmental transition to chronic infection. The continued functional expression of effector export machinery in bradyzoites, combined with stage-specific GRAs, enables *T. gondii* to maintain lifelong persistence. Understanding these mechanisms provides potential targets for therapeutic intervention against both acute and chronic toxoplasmosis.

### 3.5 The GRAs as diagnostic markers in human and animal toxoplasmosis

The GRAs of *T. gondii* represent a family of excretory-secretory antigens that have emerged as promising diagnostic markers for toxoplasmosis. These proteins, many of which are not yet fully biochemically characterized, are released from specialized organelles, and play essential roles in parasitophorous vacuole modification following host cell invasion (163). Their abundant secretion and strong immunogenicity during both acute and chronic infection phases render them ideal candidates for serodiagnosis.

Among extensively characterized antigens, GRA7 demonstrates exceptional diagnostic performance across multiple host species, achieving 81–98.9% sensitivity and 98%–100% specificity in human and canine enzyme-linked immunosorbent assays (45, 138, 139). In immunocompromised patients, recombinant GRA7 achieves 92% sensitivity and 94% specificity (140). Similarly, GRA6 is particularly valuable for distinguishing acute from chronic infections, with its N-terminal hydrophilic region demonstrating high reactivity even with low-titer sera (141). Recent investigations have identified GRA29 as a superior antigen compared to conventional tachyzoite lysate, with an area under the curve of 0.9942 (142). Additionally, GRA2 exhibits 95.8%–100% sensitivity for detecting acute infection, making it a valuable complement to existing diagnostic antigens (143, 144).

Regarding veterinary applications, GRA-based assays have been successfully adapted for multiple animal species (145, 146). GRA7-based enzyme-linked immunosorbent assay achieves excellent diagnostic accuracy in dogs, cats, and chickens (147–150). For livestock, recombinant GRA8 has been validated in goats (151). Combinations of GRA3, GRA6, and GRA7 peptides enable strain serotyping in sheep and pigs (145). Furthermore, the development of immunochromatographic tests based on recombinant antigens offers considerable potential for point-of-care testing in clinical and agricultural settings (152, 153). Notably, multi-antigen cocktails that incorporate GRA5 with surface antigens significantly enhance diagnostic sensitivity compared with single-antigen approaches (154). For veterinary applications, cross-reactivity with closely related apicomplexan parasites, such as *Hammondia* and *Neospora,* remains a diagnostic challenge, and multi-GRA cocktails may help address this by improving specificity through *T. gondii*-specific epitopes.

## 4 Conclusions and future perspectives

The GRAs represent sophisticated molecular tools that *T. gondii* uses to establish successful parasitism across diverse host species. These effectors collectively orchestrate nutrient acquisition through pore-mediated transport and host organelle recruitment, modulate immune responses by strategically manipulating NF-κB, MAPK, and interferon signaling pathways, and facilitate the developmental transition to chronic infection via tissue cyst formation.

Integration and Coordination of GRA Functions: Importantly, these three major GRA-mediated programs—nutrient scavenging, immune evasion, and cyst formation—do not operate in isolation but rather function as an integrated and coordinated system. During acute infection, pore-forming GRAs (GRA17, GRA23, GRA47, GRA72) and IVN-associated proteins (GRA2, GRA6) prioritize rapid nutrient acquisition to fuel tachyzoite replication, while immune modulatory effectors (GRA15, GRA24, TgIST) simultaneously suppress host defenses to prevent premature parasite clearance. As infection progresses, the balance shifts toward chronic persistence, with GRA proteins facilitating PVM-to-cyst membrane transition and bradyzoite-specific effectors maintaining the tissue cyst niche. This temporal coordination suggests that GRA effector deployment is hierarchically organized, with stage-specific regulatory mechanisms governing the relative contributions of nutrient-acquisition versus immune-evasion programs. The transition from tachyzoite to bradyzoite represents a fundamental shift in GRA functional priorities. While pore-forming GRAs (particularly GRA17) maintain essential roles in bradyzoite viability within tissue cysts, immune-modulatory effectors show stage-specific expression patterns, suggesting that chronic persistence requires a recalibrated balance between maintaining metabolic exchange and minimizing immune detection.

*Knowledge gaps and future directions*: Despite significant advances, several critical knowledge gaps remain that warrant focused investigation. First, the complete repertoire of GRA effectors and their precise molecular mechanisms remains incompletely defined, particularly with respect to strain-specific variations in GRA function across the diverse *T. gondii* lineages. Second, how individual GRA effectors are coordinately regulated during the tachyzoite-to-bradyzoite transition remains poorly understood. Third, the potential for functional redundancy among GRA proteins with overlapping roles—such as the multiple pore-forming GRAs or the several NF-κB-activating effectors—requires systematic analysis to determine their relative importance and hierarchical relationships. Fourth, the roles of recently identified GRAs (e.g., GRA85–88) in chronic infection biology need further characterization. Fifth, direct experimental evidence is still lacking for several proposed GRA functions, including the anti-apoptotic roles attributed to GRA5 and GRA6. Sixth, host species-specific differences in GRA–host interactions that may explain variable susceptibility across intermediate hosts remain largely unexplored.

*GRA proteins as therapeutic targets*: The remarkable functional diversity and high immunogenicity of GRA proteins position them as attractive targets for diagnostic, vaccine, and therapeutic development. GRA proteins could be targeted therapeutically through multiple approaches: (i) direct targeting of effector–host protein interactions with small molecule inhibitors, such as compounds disrupting GRA15-TRAF or TgIST-STAT1 interfaces; (ii) disruption of effector export via the MYR translocon complex, which would simultaneously impair multiple virulence mechanisms; (iii) development of pore blockers targeting GRA17/23/47/72 to impair nutrient acquisition; and (iv) immunotherapeutic strategies leveraging GRA antigens to boost host immunity, including GRA-specific T cell therapies and combination approaches with checkpoint inhibitors. Continued investigation integrating advanced proteomics, structural biology, and genetic approaches will be essential for fully elucidating GRA-mediated pathogenesis and translating these insights into effective interventions against this globally significant zoonotic disease affecting humans, livestock, and companion animals.
